# Molecular Framework of the Onset and Progression of Skeletal Muscle Aging

**DOI:** 10.3390/ijms262010145

**Published:** 2025-10-18

**Authors:** Thomas Horlem, Stephanie Rubianne Silva Carvalhal, Sandro José Ribeiro Bonatto, Luiz Cláudio Fernandes

**Affiliations:** 1 Laboratory of Cellular Metabolism, Physiology Department, Federal University of Paraná, Curitiba 80060-000, PR, Brazil; horlem@ufpr.br; 2 Laboratory of Human Molecular Genetics, Genetics Department, Federal University of Paraná, Curitiba 80060-000, PR, Brazil; stephanie.carvalhal@ufpr.br

**Keywords:** skeletal muscle, aging, metabolism, narrative review, middle age

## Abstract

Aging is a multifactorial process that progressively disrupts cellular and tissue homeostasis, affecting all organ systems at distinct rates and predisposing individuals to chronic diseases such as cancer, type II diabetes, and sarcopenia. Among these systems, skeletal muscle plays a central role in healthspan decline, yet the precise onset of its deterioration remains unclear. Most studies emphasize late-life models, overlooking the transitional phase of middle age, when initial alterations emerge. Evidence indicates that middle-aged muscle exhibits aberrant metabolism, impaired insulin sensitivity, and an early, gradual reduction in mass, suggesting that decline begins long before overt sarcopenia. This narrative review synthesizes current findings on linear and non-linear molecular biomarkers associated with the onset of skeletal muscle aging, aiming to improve early detection of muscular alterations and support the development of interventions that delay or prevent functional decline.

## 1. Introduction

Aging is a multifactorial process associated with several physiological disturbances that result in a progressive decline in cellular and tissue homeostasis. All systems of an organism are affected by this decline at different rates, contributing to the development of age-related diseases, such as type II Diabetes Mellitus or sarcopenia [1]. The importance of aging is an uptrend, as demographic studies reveal an increasing population of older adults and predict an inverted age pyramid. As a result, aging not only impacts life quality but also progressively burdens public economies like the healthcare and pension systems [2].

The last few decades have been marked by an increasing number of studies about aging and how to slow it down to prolong the healthspan of the population. However, the aging process is multifactorial and largely heterogeneous, with different rates of aging between individuals (because of genetics and environmental factors) and even between tissues of the same organism [3]. In humans, the first phenotypic alterations of the skin appear after 25 years old, in the skeletal muscle and bone after middle age (40–59 years old), and in the brain only at older ages (above 60 years old) [4,5]. Nonetheless, recent discoveries have shown that age alterations begin very early, even in low-decline tissues. For instance, some alterations associated with Alzheimer’s disease, whose pathological manifestation typically occurs after the age of 65, might begin in some individuals as soon as 30–40 years of age [6,7,8,9]. Hence, aging seems to be marked by early and cumulative alterations in the homeostasis of an organism that create a positive feedback loop that ultimately leads to frailty and diseases at older ages.

In this regard, few studies address the initial alterations related to skeletal muscle aging. Furthermore, a lack of research exists regarding the initial damage and the muscle’s response to this process [10,11]. Indeed, most of the in vivo research about skeletal muscle aging focuses on comparisons between old and young organisms, creating a gap in the field regarding mid-age alterations [10]. This creates two problems: (i) it overlooks non-linear biomarkers that return to basal values in old age after an organism initiates compensatory response mechanisms, and (ii) it presents treatment mainly as a damage-control strategy after molecular and morphological alterations are already established. These “palliative” treatments may partially promote lifespan but have a limited impact on healthspan [12,13].

**Figure 1:**
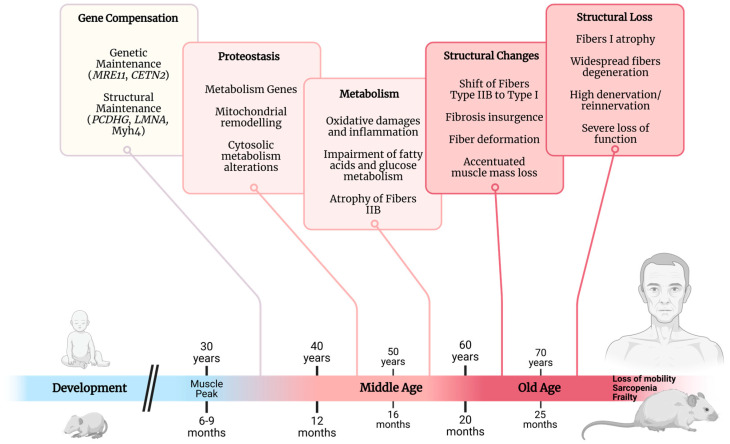
Overview of skeletal muscle aging. After the peak of muscle mass and strength is reached, gene compensation around genetic and structural maintenance is activated. Later, genetic modulation is overwhelmed, leading to proteostasis adaptations, including changes in enzyme content, mitochondrial efficiency, and signaling pathways. As aging progresses, homeostasis is eventually lost, leading to alterations in structures less energetically efficient (type IIB fibers) and a shift in the fibers to type I, which reduces ATP consumption per unit of sustained force. Ultimately, advanced age is marked by widespread loss of all fiber types and fibrosis. Note the axis break (//), representing omitted changes from the period of skeletal muscle development to allow focus on muscle aging.

Therefore, we seek to summarize and identify biomarkers indicative of the onset of skeletal muscle aging from in vivo studies on young adults and middle-aged humans and rodents in an attempt to identify some of the chronological alterations. Humans are considered middle-aged when they are between 40 and 59 years old, and rodents are considered middle-aged when they are between 12 and 20 months old—the period when metabolic parameters begin to alter (Figure 1). This review aims to contribute insights for future research seeking to prevent or delay the onset of sarcopenia—a pathologic loss of muscle mass and functionality after middle age—and contribute to answering how and when skeletal muscle decline begins.

## 2. Myonuclear Resistance to Aging

**Figure 2:**
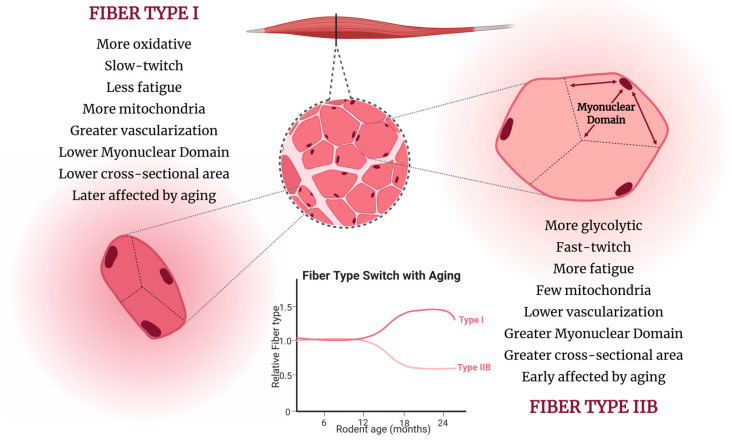
Structural and functional differences between type I and type IIB muscle fibers and their modulation with aging. Type I fibers are oxidative, fatigue-resistant, highly vascularized, contain more mitochondria, and they are less affected by aging. In contrast, type IIB fibers are glycolytic, fast-twitch, larger, and more fatigue-prone, showing earlier susceptibility to aging. The graph illustrates the age-associated shift in rodents, with an increase in levels of type I fibers and a decline in levels of type IIB fibers.

The nuclei of skeletal muscle fibers have unique characteristics relative to other tissues. For instance, muscle fibers are multinucleated, with nuclei typically positioned in the periphery close to the sarcolemma. However, exceptions occur in developmental and pathological conditions, where central nuclei are observed as a morphological biomarker for regeneration [14]. Therefore, the locations of myonuclei are tightly regulated, with the aim being to maximize the synthesis of gene products and the maintenance of its dominance region—the myonuclear domain (MND) (Figure 2). Intranuclear changes precede or accompany shifts in MND size and contribute directly to impaired gene regulation in skeletal muscle.

One of the most prominent alterations in aged skeletal muscle involves RNA production, splicing, and content, with changes—like those for the myogenic regulatory factors—ranging from 2- to 11-fold increases during aging, although protein levels remain unchanged or are even reduced [3,15]. One benefit of the overexpression of the mRNA machinery is a more developed oxidative capacity [16], along with an adaptative response to the premature loss of proteostasis in humans, mice, and rats [17]. Indeed, overall protein content loss is known to occur during skeletal muscle aging in rats starting at around 14 months-old (mo) [18,19]. In humans, a similar increase in the levels of mRNA content appears to maintain protein levels until the total mRNA declines with old age [17,20].

### 2.1. Genes Associated with Early Muscle Aging

During early aging, the genetic landscape of muscle fibers remains mostly stable, with only subtle changes emerging after 9–12 months in rodents and by around 30–40 years in humans [21]. Rare mutations in critical genes, such as *LMNA* (Pre-lamin-A/C) in Hutchinson–Gilford progeria or *WRN* (Werner syndrome RecQ helicase) in Werner syndrome, reveal the fibers’ hidden vulnerabilities, exposing nuclear architecture, DNA repair, and transcriptional control as its most sensitive domains [22]. These pathological cases act as a window into the molecular liabilities that aging gradually challenges even under normal conditions.

In this sense, genes that encode structural proteins, like *LMNA*, are among the most upregulated genes in middle-aged humans [17]. Similarly, downregulation of genes related to structural remodeling of the extracellular matrix is particularly prominent, especially in the gastrocnemius in mice and the diaphragm in rats at 9 months old [21]. Another family of genes, *PCDHG*, which encodes the protocadherin gamma, was recently found to be the main disruptor of muscle homeostasis with age [23]. *PCDHG* is persistently altered with muscle aging in fibers I and II and inversely related to muscle strength [23,24,25]. Nonetheless, studies identifying when and how *PCDHG* begins to be altered with skeletal muscle age are still lacking.

Beyond structural genes, other genes upregulated early during aging are related to genetic maintenance, such as *MRE11* and *CETN2*, which influence DNA protection by promoting the repair of double-strand breaks and nucleotide excision [17]. Deletion of genes involved in DNA repair, such as *Msh2* (MutS Homolog 2) and *Blm* (BLM RecQ Like Helicase), increases the demand for regeneration in muscle fibers; however, the regeneration process is impaired, resulting in smaller fibers [26]. Increased fibrosis and reduced strength have also been observed [26]. An early decline in DNA repair capacity is observable in other tissues too, like the liver and brain [27,28].

Another gene related to fiber protection, *CYP26B1*, shows an average upregulation of 90% in 50-year-old humans [29]. *CYP26B1* is a member of the cytochrome P450 family, which constitutes monoxygenases used to metabolize toxic substances, suggesting an adaptation to increase the elimination of toxins that accumulate with age [29]. Additionally, early changes in structural and maintenance genes indicate a reinforcement of pathways related to the protection of fiber against endogenous (metabolic subproducts, reactive oxygen species, replication errors, etc.) and exogenous (mechanical strain, toxins, environmental stressors, etc.) insults.

Unlike in other organ systems, the aging of skeletal muscle appears to be more strongly influenced by non-linear gene expression patterns, corresponding to genes whose expression increases or decreases in irregular, non-steady ways as aging progresses [30]. These alterations mostly appear during late middle age in rodents (>15 mo), when compensatory mechanisms begin to fail [30]. At the level of cytosolic products, genes for metabolic enzymes are among the genes downregulated by age starting at 9 mo in mice and rats and are linked to key genes related to signal transduction, including the signaling of tyrosine kinases, glycolysis, the tricarboxylic acid cycle, and the respiratory chain [3,17,30,31]. As a result, this period of skeletal muscle aging in rats is marked by a progressive decline in the electron transport chain, fatty acid metabolism, and oxidative phosphorylation genes, beginning in muscles rich in type IIB fibers and, later, at 18 months, in muscles rich in type 1 fibers [3,21].

Once the type IIB fibers have been impacted by aging, modifications in their fiber-specific mRNA and protein composition can be anticipated. The synthesis rates of Myosin Heavy Chain (MHC) IIA and IIx in humans have been documented to decline by 38% and 84%, respectively, compared to the muscles of young people (20–27 years) and those of middle-aged adults (47–60 years) [32]. In rats, reductions in *Myh4* mRNA and its protein (MHC IIB) are detectable from as early as 12 months old in some circumstances, occurring contemporaneously with reductions in relative muscle mass per body weight. Indeed, decreased muscle mass relative to body weight seems to precede declines in wet muscle mass and increases in atrogene expression [33].

Another gene affected by aging is *Pofut1*, a developmental gene whose expression declines rapidly by more than 50% in the first six months of life in mice and continues to decay progressively until the animals reach old age [34]. The main function of *Pofut1* revolves around post translational modification of Notch Receptors with glycosylation of serine or threonine residues. *Pofut1*’s role in development and aging is unclear: its downregulation is linked to hypertrophy and muscle maturation in very young mice, but at an advanced age, the effects of its decline are more deleterious, leading to fiber atrophy, muscle function decline, impaired regeneration, and altered neuromuscular junctions in knockout mouse models [34,35]. In other tissues, *Pofut1* expression is linked to hepatic fibrosis containment, coronary protection, and cellular senescence, although it is also upregulated in cancer [36,37,38,39].

### 2.2. Transcript Factors Drive Muscle Aging

Many gene alterations are regulated or coordinated by transcription factors, which are among the earliest molecular elements to respond to aging in skeletal muscle. Overall, transcription factors play a central role in orchestrating early gene responses to aging in muscle—beginning as early as 12 months in rats and 30 years in humans—as they attempt to buffer homeostatic imbalance [3,30].

One example is the downregulation of the expression of the Musculoaponeurotic Fibrosarcoma (MAF) family of transcription factors, which is highly expressed in the skeletal muscle and leads to compromised maintenance of glycolytic metabolism [40]. The MAF family directly regulates type IIB fibers at the transcription level in structural genes, like *Myh4*, which encodes MHC IIB, and metabolic genes, like *HK1* (hexokinase) and *PFKM* (phosphofrutokinase) [40]. Moreover, MAF is linked to the activation of PI3K/Akt signaling, calcium homeostasis, and type IIB fiber maintenance upon denervation [41,42]. Nonetheless, its role in myoblast proliferation is not clear, with divergent reports about MyoG levels when MAF is suppressed or overexpressed [40,41].

**Figure 3:**
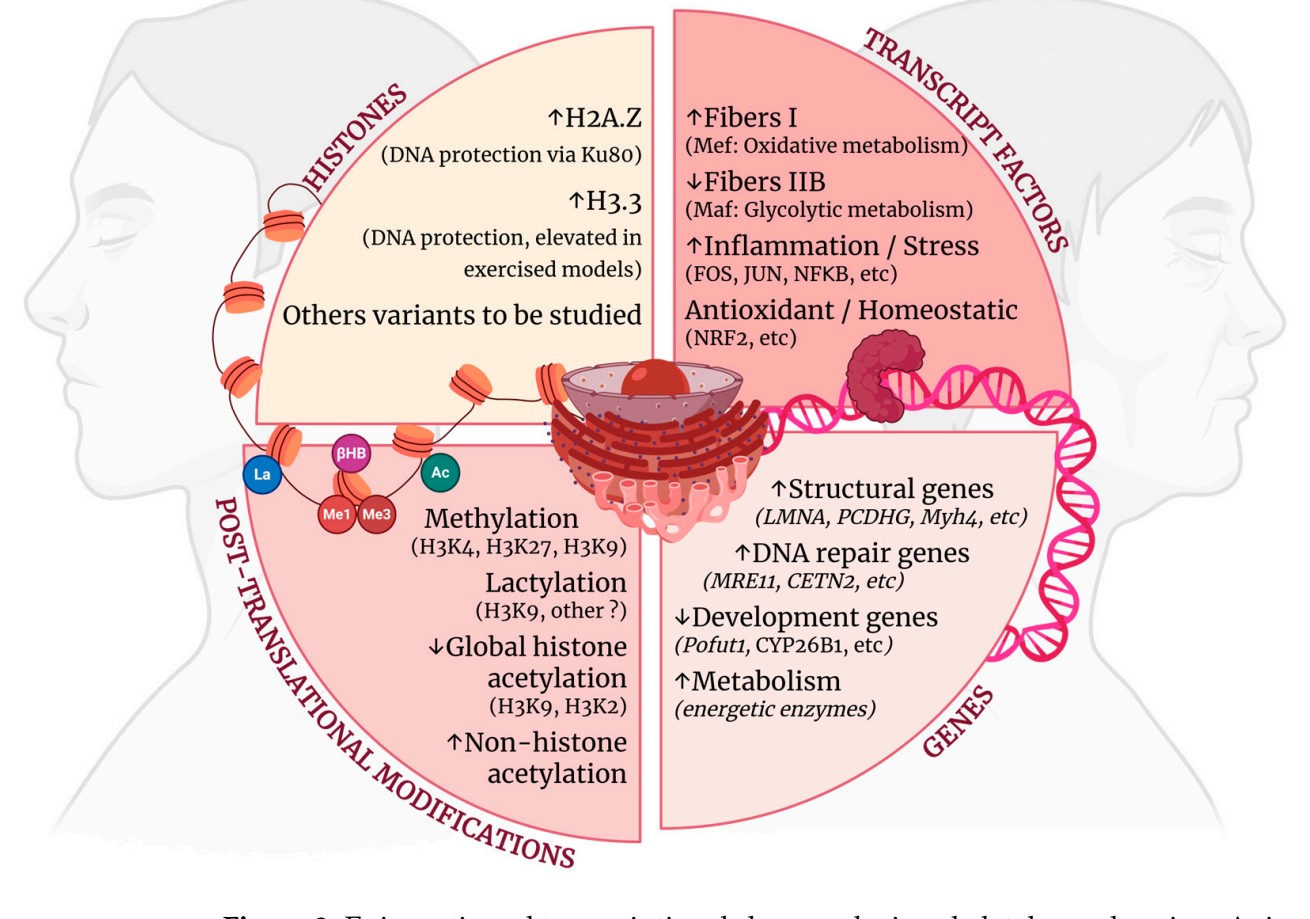
Epigenetic and transcriptional changes during skeletal muscle aging. Aging muscle displays alterations in histone variants (H2A.Z and H3.3), post-translational modifications (methylation, acetylation, and lactylation), and gene expression programs regulating structure, repair, metabolism, and development. Transcription factors further shift fiber type composition, enhance stress/inflammatory signaling (FOS, JUN, and NF-κB), and impair antioxidant defenses (NRF2), collectively contributing to functional decline.

MAF also interacts with other transcript factors, like the myocyte enhancer factor 2 (MEF2) family, which exhibits decreased expression as MAF is overexpressed [40]. MEF2D exhibits a significant and linear rise throughout human life, which is inversely correlated with MAF levels as skeletal muscle ages [17]. The MEF2 transcript factor is related to increased oxidative capacity in muscles, enriching gene patterns towards type I fiber differentiation (Figure 3) [43]. Moreover, the MEF2 family of transcription factors is also fundamental for muscle regeneration, underpinning a compensatory mechanism with its upregulation throughout one’s life [44,45,46].

A different group of transcription factors modified during this period in rats is regulated by FOS and JUN, which heterodimerize to create the transcription factor AP-1. AP-1 is recognized for its physiological role in the activation and proliferation of satellite cells as well as in facilitating the adaptation of fibers to mechanical stress [47]. However, chronic activation of AP-1 with skeletal muscle aging is connected to upregulation of inflammatory genes, oxidative stress, and fibrosis—all characteristics that are well defined in muscle aging in rodents and humans [24,48].

Other transcription factors associated with the inflammatory response and oxidative stress are also commonly affected by skeletal muscle aging. For instance, fiber damage is linked to NF-ΚB activation, which raises pro-inflammatory cytokine expression and secretion [49]. After one reaches middle age, NF-ΚB activity is increased, contributing to a chronic low-grade inflammatory environment in the muscle that continuously accelerates muscle decline even at older ages [49]. Transcriptomic data shows linear and pan-tissue disruption of NF-ΚB in mouse, rat, and human models [3,50]. Its increased activation may be seen as early as 12 months-old in rat models [51]. In the context of cancer cachexia, NF-ΚB is also linked to fiber atrophy via immunomodulation of macrophages that have infiltrated muscle, suggesting there are similar mechanisms in muscle aging once macrophage and NF-ΚB levels have both increased with old age [52].

Unsurprisingly, antagonists of NK-ΚB are also altered with muscle aging. This is the case for nuclear factor erythroid 2-related factor 2 (NRF2), which controls fibers’ defense against oxidative insults and low-grade basal inflammation [53]. Its deregulation is central to the aging phenotype in satellite cells and linked to glutathione depletion, ultimately leading to a loss of regenerative capacity [53]. In the context of fibers, knockout of NRF2 in mice accelerates the loss of muscle mass and strength. Moreover, knockout of NRF2 hinders mitochondrial biogenesis, as seen via SDH staining and PCG1α protein and mRNA decline in mice that were 11–13 mo [54].

The impact of transcription factors is significant not just at the onset of muscle aging but also throughout its course. For instance, knockout of ATF4—associated with atrophy in malnutrition—helped to improve muscle quality and preserve muscle strength and mass in elderly mice [55]. Similarly, the knockout of forkhead box O (FoxO)—which exhibits increased activity in sarcopenia and promotes the generation of proteolytic genes and atrophy—mitigated the loss of muscle strength and increased mitochondrial oxidative phosphorylation capacity by 10–20% in 22 mo mice [56]. Overall, the transcription factor alterations that occur with aging also underlie conserved metabolic and mitochondrial alterations across different species, including mice, rats, and humans [3].

### 2.3. Histone Adaptations

The aging of myonuclei is not just related to alterations in gene expression during early middle age, as discussed above, but also to histone and epigenetic changes that directly aggravate or attenuate the phenotype of aging [57,58,59]. One of these alterations is related to the variant H2A.Z, which is already linked to cognitive decline and whose presence is greater in muscle aging [60,61]. However, the increased presence of H2A.Z in skeletal muscle appears to be an adaptation to aging rather than a causative factor: its knockout accelerates muscle aging, leading to the atrophy of type II fibers, disturbed metabolism, mitochondrial defects, and neuromuscular alterations [58].

One of the benefits of H2A.Z in muscle aging may be related to its role in DNA protection. It appears to interact with Ku80, a protein that is essential for telomere maintenance and the repair of double-strand breaks [58,62]. In this context, the lack of one *Ku80* allele in heterozygous knockout mice accelerated muscle aging at 6 mo, as shown by a fiber type population profile similar to that of physiologically aged mice (18 mo) [62]. Other muscle features of heterozygous Ku80 mice include impaired regeneration via increased p53 content, reduced phosphorylation of Akt, increased mitochondrial density, and increased DNA damage [62]. Overall, this information suggests that even slightly lower levels of Ku80 protein at baseline can compromise muscle homeostasis, even in young individuals.

Another histone variant, H3.3, shows a linear increase in expression in mice 2 to 18 months old. However, an even stronger expression of H3.3 can be observed in mice that have exercised, suggesting that H3.3 may serve as a potential counterbalance to aging and a biomarker for muscle health [63]. While aging does not significantly alter the levels of the H3.1 and H3.2 histone variants, it does notably increase the ratio of these histones compared to the content of H3.3 [63]. Interestingly, the loss of the H3.3 histone variant is associated with impaired myogenesis in certain myopathies, such as dermatomyositis [64].

Many other histone content changes may occur in muscle aging but are not properly studied in the context of physiological muscle aging. For instance, the macro histone mH2A1’s expression is related to improved glucose metabolism, reduced adipogenesis, inhibited basal poly-ADP ribose polymerase 1 (PARP-1) activity, and reduced nuclear NAD+ consumption, features that directly impact muscle aging [65,66]. However, to the best of our knowledge, the expression of mH2A1 in skeletal muscle during middle age has not been investigated.

### 2.4. Post-Translational Changes

Not only does histone content change with muscle aging, but it also undergoes post-translational regulation. The two main types of post-translational modifications are methylation and acetylation. Together, these modifications affect how histones interact with DNA, either promoting or repressing the transcription of genetic information. As people age, these processes change significantly. In fact, the changes in methylation patterns associated with aging form the basis for some of the most reliable biological age estimates, often called aging clocks [67].

Histone methylation dynamics in muscle fibers are notably sensitive to aging, with changes already evident between 2 and 13 months in mice and rats [33,63,68]. Examples of age-associated methylation changes in skeletal muscle include monomethylation of H3K4 and H3K27, which increases linearly with age [63,69]. Among the H3K4 methyltransferases, Mll2 plays a crucial role in maintaining glucose metabolism. Impairments in Mll2 are linked to insulin resistance, highlighting the connection between changes in histone methylation and metabolic dysregulation in aging muscle [70]. Similar alterations in metabolism and methylation have been observed with the overexpression of DNA methyltransferase 3, which can lead to damage in type IIB muscle fibers and a shift in fiber populations towards type I fibers [71]. Importantly, the relationship between metabolism and methylation is bidirectional: increased insulin sensitivity is associated with higher AMP levels, which, in turn, correlate with reduced H3K4 methylation [72].

Stronger effects of age-associated methylation are observed with the histone trimethylation of facultative heterochromatin sites, a process that increases in potency during aging [69]. Notably, the repressive trimethylation of H3K27 is exacerbated early in skeletal muscle atrophy models, a process preceding the global DNA hypermethylation that contributes to transcriptional downregulation during atrophy [73]. A central mediator of exacerbated methylation is the Polycomb Repressive Complex, particularly its catalytic subunit EZH2, whose activity progressively increases with age [74]. Consistently, methylation cluster analyses across multiple timepoints in aging mice revealed an enrichment of transcription factor binding sites for MTF2 (Metal response element-binding transcription factor 2), a critical mediator of Polycomb recruitment, as well as for EZH2 itself after 15 months in mice [67,75]. This finding is further supported by a meta-analysis of skeletal muscle epigenomic aging, which showed that numerous Polycomb targets become increasingly methylated with age in several tissues, including skeletal muscle [74,76].

Parallel with the increase in facultative heterochromatin, there is a corresponding loss of constitutive heterochromatin, as indicated by the levels of H3K9 trimethylation. This loss of H3K9 leads to significant nuclear disorganization and genomic instability in vitro [77]. In experiments involving knockout mice, this loss has been associated with muscle atrophy, premature epigenetic aging, and a reduced lifespan [57,77]. Notably, diminished H3K9 trimethylation not only compromises the integrity of heterochromatin but also shifts the chromatin landscape toward a more permissive state [77]. In this context, histone acetylation emerges as a crucial epigenetic mechanism that influences gene accessibility and transcription during aging [78].

Despite the theoretical relevance of histone acetylation in aging, primary data on skeletal muscle remains scarce. Studies on rats suggest global hypoacetylation (H3, H3K9ac, and H3K27ac), while analyses of non-histone proteins of the gastrocnemius and quadriceps muscles revealed elevated acetylation up to 3-fold by 12 mo that remained continuously elevated after 20 mo in mice and rats [33,79]. The central regulators of acetylation in the fibers are sirtuins (SIRT) and histone deacetylase (HDAC), which control several metabolic and genetic pathways [78]. In this vein, levels of SIRT1 5 and 6 were found to have increased within 12 mo in mice, indicating initial compensatory mechanisms were employed to try to contain the excessive non-histone acetylation in the skeletal muscle [33,79].

Nonetheless, deacetylation reactions in muscle fibers become impaired. This impairment occurs because the activity of an essential cofactor, NAD+, is reduced in aged fibers [80]. Consequently, treatments aimed at restoring NAD+ levels are among the latest strategies used to slow down muscle deterioration associated with aging [80]. Other treatment options include resveratrol, which enhances SIRT1 activity and improves overall deacetylation, and butyrate, which inhibits HDAC and increases histone acetylation [81,82].

The interplay between histone modifications and muscle metabolism extends beyond methylation and acetylation. One metabolic product commonly generated in skeletal muscle during intensive glycolysis is lactate. Lactate promotes lactylation of histones and actively promotes myogenesis through H3K9 lactylation-mediated upregulation of genes such as *Neu2*, which supports satellite cell differentiation and muscle regeneration [59,83]. During muscle aging, histone lactylation also plays a protective role by antagonizing cellular senescence and modulating aging-related pathways [59].

Similarly, another important metabolite involved in muscle physiology is β-hydroxybutyrate, which is produced in the liver during fasting and caloric restriction. High levels of β-hydroxybutyrate serve as a powerful energy source when glucose is not available. Additionally, this metabolite leads to the β-hydroxybutyrylation of histones in skeletal muscle [84]. In accordance with the advantages of caloric restriction for aging, β-hydroxybutyrylation of histone H3K9 has been shown to enhance muscle function and metabolism [84]. This occurs by promoting the upregulation of mitochondrial genes in both in vitro and in vivo models while also suppressing the expression of FOXO-dependent atrogenes [84,85].

It is important to highlight the differences in muscle responses to aging among different species. While muscle aging in rats and humans exhibits a more complex and dynamic genetic pattern, this complexity is not observed in mice, which are the most used models in studies [3,21]. In fact, mouse models appear to be less susceptible to alterations in metabolism and mitochondrial function pathways during aging [21]. This discrepancy may contribute to the divergent results between pre-clinical and clinical studies.

## 3. Homeostatic Impairment

As discussed above, the interplay between metabolism and aging is tightly regulated in skeletal muscle aging. Indeed, homeostatic loss and aberrant metabolism are hallmarks of muscle dysfunction. Several studies have shown that in old age, mitochondrial enzymes suffer an alteration in activity, directly impacting cell metabolism and posing a risk of mobility decline [86,87]. Early in aging, an apparent rise in mitochondrial content has been observed; it is largely attributed to impaired autophagic clearance and compensatory mitochondrial biogenesis in response to energy demands. This is frequently associated with a moderate elevation in the levels of reactive oxygen species (ROS), which, at physiological levels, act as hormetic signals for stimulating mitochondrial turnover and stress-resistance pathways. However, when ROS production becomes chronic or excessive, these same signals promote oxidative damage, destabilizing proteostasis and impairing muscle fiber integrity. Although these metabolic changes are seen in sarcopenia, they tend to occur much earlier than other sarcopenic symptoms [3,87].

### 3.1. Mitochondrial Adaptations

The first changes in mitochondria can be observed not through damage to mitochondrial DNA (mtDNA) but rather via the activity of their structural and functional proteins [88]. Molecular analyses, particularly transcriptomic studies, show that alterations in oxidoreductase pathways and energy metabolism begin around the age of 30 in human skeletal muscle [3]. After this age, initial functional changes become evident in muscle activities, such as increased recovery time required after exercise and reduced minimum oxygen saturation [89,90]. More significant functional alterations, such as those measured by the widely used grip strength test, typically manifest later, between middle and old age.

Decreased levels of oxidative phosphorylation—and consequently lower ATP levels—are well-established biomarkers of muscle aging and sarcopenia [29,86]. In fact, changes in the electron transport chain are among the few pathways in muscle that are consistently altered with age across various species, such as worms, flies, mice, rats, and humans [21,29]. Some studies indicate that alterations in oxidative phosphorylation also occur before the loss of insulin sensitivity in skeletal muscle during aging [91,92].

Optimal functioning of the mitochondrial electron transport chain is essential not only for enhancing electron flow during oxidative phosphorylation but also for maintaining the protein content of the machinery that produces ATP [93]. Research indicates that the expression levels of most proteins from complexes II, III, IV, and V remain unchanged during the early stages of skeletal muscle aging [94,95,96,97,98]. The only complex particularly affected by middle age appears to be Complex I, whose protein levels and activity are reduced [95,98]. This likely reflects the oxidative capacity of an organism, as Complex I levels remain higher in older individuals with naturally greater oxidative capacity [16]. Similarly, individuals who increase their mitochondrial capacity through regular exercise also exhibit higher Complex I levels, emphasizing the importance of this complex for maintaining energy homeostasis [16].

Not only are some intra-mitochondrial proteins related to oxidative phosphorylation affected by muscle aging, but structural proteins involved in mitochondrial morphology are also impacted. Indeed, alterations in mitochondrial morphology seem to precede overt muscle aging, as evidenced by an increased presence of cristae in skeletal muscle mitochondria during peak adulthood in rodents [99]. Similarly, cardiac muscle in mice shows reductions in mitochondrial size and area but also an increase in the number of mitochondria during early aging (12 mo mice), suggesting that mitochondrial adaptations occur across different muscle types [100].

At an advanced age, skeletal muscle mitochondria decline in density and/or total number [87,96,101], while their size and/or area tend to increase as a compensatory mechanism [87,102,103,104]. Over time, cristae patterns also change, leading to reduced cristae density, particularly in intermyofibrillar mitochondria [101,102]. While these changes in mitochondrial morphology may reflect a peak stage of muscle development, they could also represent a compensatory response aimed at increasing the surface area of the electron transport chain (ETC) to sustain oxidative phosphorylation during the early stages of aging [105]. Regardless of the underlying cause, the initial temporary increase in mitochondrial numbers and cristae, followed by functional decline, aligns with the Hyperfunction Theory of Aging, which proposes that aging is driven by the persistent activity of biological programs beyond their optimal period [106].

Mitochondrial cristae remodeling also coincides with changes in lipid metabolism. Higher cristae density has been inversely associated with the accumulation of lipid droplets within muscle fibers, suggesting that mitochondrial efficiency may act as a buffer against ectopic lipid deposition and impaired glucose metabolism [101]. Indeed, aging skeletal muscle exhibits an inability to shift substrate consumption from lipid to glucose upon stimulation, revealing impaired metabolic flexibility with age [107].

### 3.2. Mitochondrial Dynamics

The protection of mitochondrial functionality and mitochondrial DNA (mtDNA) is closely regulated by the dynamics of this organelle, including its biogenesis, fission, fusion, and autophagy [78,108]. These processes tend to be disrupted during the aging of skeletal muscles [78]. This regulation is connected to mitochondria-shaped factors, which encompass fusion and fission proteins that play a crucial role in maintaining mitochondrial functionality and quality [108].

One well-known fusion protein is Mitofusin-2 (Mfn2), whose expression begins to decline in mice at around 12 months of age [87,94,109] and in rats at approximately 18 months [102]. However, some studies suggest that there may be a temporary increase in either *Mfn1* or *Mfn2* expression prior to this decline in skeletal muscle during the aging process [87,94]. The reduction in Mfn2 levels in older age has been associated with a decrease in the number and size of mitochondrial cristae, while the overall number and density of mitochondria may increase [110]. A similar decline in another mitochondrial fusion protein, Mitochondrial Dynamin Like GTPase (Opa1), begins at around 12 months of age in mice [87,100], resulting in a corresponding reduction in mitochondrial size [87,100,110].

Alterations in fusion proteins are associated not only with changes in mitochondrial shape but also with abnormalities in fibers and tissues. These abnormalities include inefficient cellular growth, impairment of the respiratory chain, increased production of reactive oxygen species (ROS), exhaustion of stem cells, and defective fiber regeneration [111,112]. Furthermore, the effects of systemic ablation of Opa1 extend beyond muscle sarcopenia, contributing to an aging phenotype in an organism. This includes symptoms such as white hair, denervation, and liver steatosis [113]. Additionally, the deletion of *Opa1* in newborn mice severely disrupts development and can lead to death [113].

Other factors that influence mitochondrial morphology include fission proteins such as Dynamin-related protein 1 (Drp1) and Mitochondrial fission 1 protein (Fis1). Impairments in the content and activity of Drp1 and Fis1 significantly affect the macroautophagy of damaged mitochondria, which is linked to a loss of proteostasis and the promotion of insulin resistance [114]. Furthermore, physiological levels of IL-6, which are stimulated by exercise, can increase the protein content of Drp1 and Fis1 [115]. In mice, an early decrease in the expression of these proteins in hindlimb muscles occurs between 12 and 18 months [87,94,98].

It is important to note that changes in fission proteins do not always have negative correlations with muscle fusion proteins. For example, research has shown that the expression of the Fis1 protein decreases as Opa1 levels increase [94]. Similarly, a reduction in *Drp1* mRNA expression was observed alongside a decrease in *Opa1* mRNA levels [87]. There were also instances where Drp1 protein levels fell while Opa1 levels remained unchanged [98]. This illustrates how various molecules can help mitochondria adapt to early aging stress, with the overall effects dependent on a balance of conflicting or synergistic signals.

The MICOS (Mitochondrial Contact Site and Cristae Organizing System) complex plays a crucial role in regulating the internal and external morphology and function of mitochondria. Research indicates that alterations in the proteins that make up this complex are closely associated with aging [102]. The deletion of the MICOS complex is linked to a reduction in mitochondrial capacity and a decrease in both basal and maximum oxygen consumption rates in knockout myotubes [116]. Structurally, loss of the MICOS complex leads to an increase in the number of mitochondria, a reduction in size, and the promotion of abnormal internal cristae [116].

### 3.3. Insulin Sensitivity and Glucose Intolerance

Individuals aged 40 to 50 experience significant changes in their skeletal muscle fibers, particularly outside the mitochondria. By this age, alterations in proteostasis have already begun, prompting the activation of various compensatory mechanisms in the sarcoplasm to maintain muscle functionality. Certain studies suggest that compensatory responses are associated with an increase in insulin receptor content but also a subsequent decline in insulin receptor substrate (IRS) levels [117,118,119,120,121]. Additionally, total Glucose Transporter 4 (GLUT4) levels rise with age, becoming evident as early as 12 months of age in mice and 17 months of age in rats [119,121]. However, despite these adaptations, the efforts of these fibers are insufficient to prevent the eventual decline in insulin sensitivity over time. This decline is reflected in higher basal glycemia and reduced peripheral glucose uptake during old age [88,119,121,122,123].

**Figure 4:**
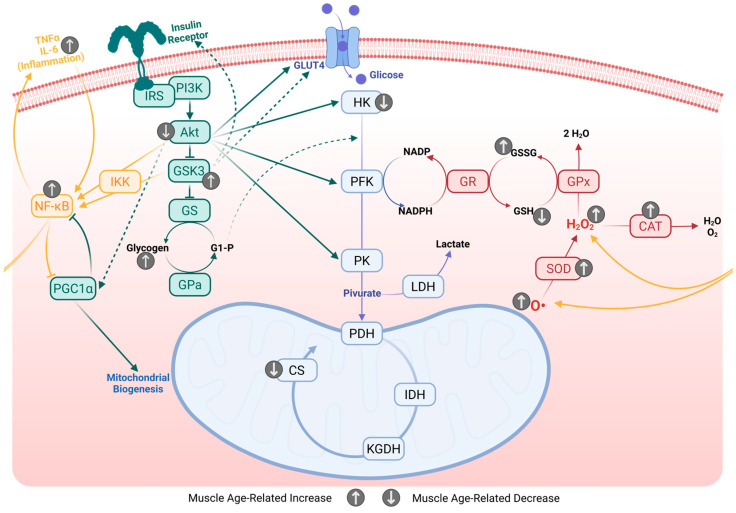
Skeletal muscle homeostatic decline impacts glycolysis, oxidative phosphorylation, inflammatory signals, and oxidative residues. The glycolysis rate-limiting enzymes, namely, hexokinase (HK), phosphofrutokinase (PFK), and pyruvate kinase (PK), are all influenced by the PI3K-Akt pathway. Pyruvate can be directed to oxidative phosphorylation, where the rate-limiting enzymes are pyruvate dehydrogenase (PDH), isocitrate dehydrogenase (IDH), alpha-ketoglutarate dehydrogenase (KGDH), and citrate synthase (CS). Pyruvate may be diverted to lactate production by lactate dehydrogenase (LDH) if the former is present in excessive levels, e.g., during intense exercise or when there is inefficient oxidative phosphorylation. The PI3K-Akt pathway, activated by insulin receptor signaling, also inhibits glycogen synthase kinase 3 (GSK3), which inhibits glycogen accumulation by glycogen synthase (GS). Other proteins affected by PI3K-Akt include IκB kinase (IKK) and Nuclear Factor Kappa B (NF-κB), both of which are related to pro-inflammatory cytokine production and release. Inflammatory signaling and energetic pathways are intrinsically linked by oxidative production and its neutralization by superoxide dismutase (SOD), catalase (CAT), and glutathione peroxidase (GPx), the last of which relies on reduced glutathione (GSH), which is recycled from its oxidized form (GSSG) by glutathione reductase (GR). Arrows colors: red for redox metabolism pathways; blue for glycolysis and tricarboxylic acid cycle pathway; yellow for inflammatory pathways; green for other metabolic proteins.

Under physiological conditions, GLUT4 on the sarcolemma regulates glucose uptake through the action of phosphoinositide-dependent kinase 1 (PDK1) and 2 (PDK2) and Akt. Both of these kinases are activated by phosphatidylinositol trisphosphate (PIP3), which is produced by phosphatidylinositol 3-kinase (PI3K), a pathway that is downstream of the insulin-stimulated IRS-1 protein (see Figure 4). A knockout rat model of IRS-1 exhibited impaired development, muscle atrophy, glucose intolerance, insulin resistance, and an increase in IRS-2 levels as an adaptive response involving PI3K [120].

As muscles age, the central activity of Akt is disrupted as early as 13 months in rats and mice [51,97,122]. Similarly, in humans, insulin-activated Akt2 levels decrease to 40% compared to young subjects, which is associated with a 25% reduction in the rate of peripheral glucose uptake [107]. Furthermore, animal models that lack Akt have shown that disruption of Akt signaling promotes sarcopenic phenotypes, including reduced glycolytic fibers and muscle mass [122].

Reduced Akt activity leads to apoptosis, which coincides with the upregulation of apoptotic and myogenic pathways that occur later in response to age-related decline in muscle fibers [21]. Additionally, one of Akt’s targets is FoxO, a transcription factor that plays a crucial role in protein degradation and the loss of muscle mass and antioxidant capacity. Inactivation of FoxO can counteract the effects of Akt loss, potentially enhancing muscle metabolism and strength [56,122].

Finally, the enzymes PDK1/2 and Akt play a crucial role in the regulation of glucose storage by inhibiting glycogen synthase kinase 3 (GSK3), a repressor of glycogen synthase. When upstream signaling is impaired, muscle fibers can more easily deplete their glycogen reserves due to reduced inhibition of GSK3 [124]. Additionally, GSK3 helps muscle fibers effectively utilize available energy by activating the protein synthesis machinery through direct interaction with mTOR and indirect interaction with eIF2-B [125]. However, this protein synthesis machinery begins to be downregulated as early as 16 months in rodents when disruptions in this pathway occur [51,121].

### 3.4. Energetic Deviations from Homeostatic Metabolism

After midlife, alterations in proteostasis and mitochondrial function lead to progressive impairment of proteins involved in glucose and lipid metabolism. This contributes to reduced glycolysis and stimulates cellular senescence, carbonyl stress, and other maladaptive responses [96,107,126,127]. In older rodents (more than 18 months old), fatty acid metabolism is reduced in the gastrocnemius, soleus, quadriceps, plantaris, extensor digitorum longus (EDL), and tibialis anterior muscles [3,21,30,102]. Additionally, an accumulation of intramyocellular lipid droplets occurs early and is associated with the phenotype of sarcopenia [3,96,127].

Lipid transport proteins, such as CD36 and fatty-acid-binding protein 3 (FABP3), play a crucial role in metabolic shifts by coordinating glucose homeostasis, SR stress, and proteostasis maintenance [128]. Research shows that the genetic deletion or pharmacological inhibition of these proteins in aged models is associated with improved metabolic flexibility and partial preservation of muscle morphology [96,128]. Likewise, mitochondrial import of fatty acids via carnitine palmitoyltransferase 1B (CPT1B) also declines with age in terms of both expression and activity [129]. Furthermore, lipid enzymes are dysregulated with aging; for instance, glycerophosphocholine phosphodiesterase 1 (GPCPD1) shows significant disturbances in aged muscle, leading to impaired insulin signaling [130]. These findings support the hypothesis that excessive lipid influx and oxidation contribute to the deterioration of muscle associated with aging in its early stages.

Metabolic substrate impairment in muscle aging is reflected in glycogen content and utilization. Studies using electron microscopy and biochemical assays have shown that glycogen content tends to increase with age, particularly in glycolytic fibers (type IIB) [131,132,133,134,135,136]. Other studies indicate that while glycogen content may not significantly change, there is an increased rate of glycogen depletion after mobilization. This suggests that older muscles must consume larger amounts of glycogen to meet the same ATP demands during stimulation [137,138].

The consequences of metabolic inflexibility extend beyond just energy deficits. Aging muscle exhibits increased levels of markers of cellular senescence, particularly cyclin-dependent kinase inhibitor p16, whose expression is upregulated [5,139]. This alteration is closely linked to chronic low-grade inflammation, which promotes abnormal paracrine signaling, which has systemic effects [127]. These characteristics closely resemble the impacts of long-term exposure to a high-fat diet (HFD), indicating a potential mechanistic connection between impaired lipid metabolism, mitochondrial dysfunction, and pro-senescent signaling during muscle aging [140].

The effects of a high-fat diet (HFD) on aged muscle highlight the metabolic inflexibility associated with muscle aging. Key enzymes involved in beta-oxidation, such as very-long-chain acyl-CoA dehydrogenase (VLCAD) and medium-chain acyl-CoA thiolase (MCKAT), as well as those involved in glycolysis, including enolase 3 (ENO3), phosphoglycerate kinase 1 (PGK1), phosphoglycerate mutase 2 (PGAM2), phosphoglucomutase 1 and 2 (PGM1/2), and pyruvate kinase muscle isozyme 1 (PKM1), show a decreased response to HFD [129]. Furthermore, adhering to an HFD during middle age leads to reduced insulin sensitivity and muscle mass, promotes satellite cell exhaustion, and contributes to the development of various sarcopenic traits [140].

Notably, in the context of healthy muscle, like in 1–3 mo mice, a HFD diet’s effects on body weight and muscle functionality can be completely reversed after one to five months of adherence to a normal diet [68]. Furthermore, young and healthy muscle triggers adaptive mechanisms with respect to HFD that remain active for longer periods, even after a HFD is no longer adhered to. These lifelong residual responses (up to 10 months) include upregulation of Ucp3, Pdk4, and Zmynd17—genes related to higher expression of histone H3.3 and an improved response to insulin [68].

### 3.5. Antioxidant System

After the metabolic alterations occurring during the beginning of muscle aging have occurred, levels of oxidative stress and inflammatory responses begin to rise. It appears to happen at around 40 years of age in humans, when the decline in muscle mass slowly starts to become more evident [3,141]. The main defense mechanism against oxidative stress in skeletal muscle fibers relies on the glutathione system and Coenzyme Q (CoQ).

The glutathione system is an effective mechanism for preventing excessive oxidative stress via respiration, which can damage lipids (lipoperoxidation) and proteins (oxidation and carbonylation). These damaging processes tend to accumulate with age and are negatively correlated with the antioxidant potential of a cell [142]. Significant changes in lipoperoxidation occur between 11 and 19 months of age, with observable changes already noticeable at around 14 months in rats [18] and mice [94]. Additionally, levels of 4-HNE and the fluorescent ROS sensor CM-H2DCFDA are higher at around 12 months compared to 3–6 months [79,97,143]. Ultimately, the imbalance between cellular defense mechanisms and the oxidative stress produced has a profound impact on cellular metabolism and mitochondrial maintenance. This leads to a decline in homeostasis in fibers and satellite cells [53,144].

Several studies have shown disruptions in the complex network of the glutathione system in middle-aged animal models. For instance, glutathione peroxidase, which plays an essential role in neutralizing free radicals by oxidizing reduced glutathione, shows increased activity in both early- and late-middle-age rats [18,19] and mice [82]. The oxidized glutathione produced in this process is later recycled by glutathione reductase, whose activity also increases concurrently (Figure 4) [18,19].

Although the overall glutathione levels remain constant with the advancement of muscle aging, the activity of γ-glutamylcysteine synthetase and glutathione synthetase—enzymes responsible for the initial stages of glutathione synthesis—first rises in rats aged 6 to 15 months and subsequently decreases between 15 and 18 months [18,145]. This suggests high protein turnover is employed as an initial adaptive response, with an elevated energetic cost, aimed at maintaining muscle homeostasis until the fibers can no longer afford it or is overwhelmed by the oxidative burden. This coincides with unbalanced levels of reduced and oxidized glutathione during middle age, when depletion of reduced glutathione begins to be observable between 12 and 19 months in both mice and rats [53,145]. Finally, glutathione S-transferase, an enzyme involved in detoxification within muscle fibers, shows increased activity during this same period in the soleus and vastus lateralis muscles of rats [18,19].

Coenzyme Q (CoQ) is another protective mechanism against oxidative damage in the mitochondria, acting as an electron shuttle while simultaneously regulating the permeability of mitochondrial pores and ions. This control is inextricably linked to the mitochondrial capacity for oxidative phosphorylation. Some studies have found that CoQ levels are lower in older age groups, a feature that has been linked to skeletal muscle atrophy and sarcopenia [78]. However, CoQ seems to have a very dynamic presence in skeletal muscle during aging, with the total CoQ content increasing by up to 1.8-fold in middle-aged mice (12 mo) relative to that in young mice (6 mo) and yet being almost restored to baseline levels during old age (21 mo mice) [146]. Curiously, this fluctuation seems to be more linked to CoQ9 than CoQ10, given that the levels of the latter remained high [146].

Similar increasing values of CoQ until middle age are observable in samples of plasma, skin surface lipids, and the brain, liver, and kidneys [147,148,149,150] from rodents and humans, indicating a systemic response to mitochondrial stress at the start of organismal aging. CoQ10 supplementation has also been linked to functional improvements, at least in the motor cortex of the brain, where neural activity was found to be increased in 15-month-old mice [151,152].

Supplementation with CoQ also has protective effects on muscle, resulting in increased mitochondrial respiration, muscular strength, nuclear organization, and myogenesis [153]. Similarly, one study involving young mice acutely injured with burns found that treatment with CoQ10 rescued skeletal muscle alterations like those found in aged models (decreased oxidative phosphorylation, impaired insulin sensitivity, loss of mitochondrial cristae, oxidative distress, and inflammation) [154]. As a result, elevated CoQ levels until middle age appear to help skeletal muscle adapt to aging and sustain homeostatic function.

In addition to glutathione and CoQ, other enzymes such as catalase and superoxide dismutase play a crucial role in protecting muscle fibers and mitochondria from oxidative damage. Research indicates that catalase levels gradually increase in rodents from the age of 12 to 19 months, peaking after 20 months [18,19,82,155,156]. In rodent models, the mRNA levels of superoxide dismutase (*Sod1* and *Sod2*) decrease with muscle aging. However, the content and activity of CuZnSOD (the cytosolic isoform encoded by *Sod1*) and MnSOD (the mitochondrial isoform encoded by *Sod2*) increase after early middle age [18,82,157,158,159,160]. These increases are observed in several types of muscle, as shown by the heightened total superoxide dismutase levels in the biceps brachii, sternomastoideus, soleus, plantaris, and gastrocnemius muscles of 12-month-old rats [160].

Notably, heterozygous knockout of *Sod2* in mice did not accelerate muscle aging, but it did increase mtDNA damage [161]. Although mtDNA damage does not always impair insulin signaling or oxidative phosphorylation, compensatory metabolic responses to such damage involve transcription factors (NRF2, PPARγ, and Sirtuins) and higher levels of proteins from the respiratory chain and involved in mitochondrial protection, including CuZnSOD and MnSOD [88]. MnSOD expression reinforces PI3K/Akt signaling, enhances sarcoplasmic reticulum Ca^2+^-ATPase activity, and indirectly preserves mitochondrial DNA, thereby supporting muscle mass and functionality [162,163,164]. Interestingly, while muscle-specific deficiency of MnSOD or CuZnSOD does not lead to significant muscle-mass loss, deletion of neuronal CuZnSOD indirectly accelerates muscle aging and precedes sarcopenia in knockout mice by disrupting the interplay between muscle and motoneurons [165,166,167].

### 3.6. Ionic Homeostasis

One of the main functions of mitochondria, together with the sarcoplasmic reticulum (SR) and sarcolemma, is to control the influx and efflux of ions in fibers during contraction, a task that is fundamental for fiber depolarization and optimal contraction. Homeostatic anomalies in skeletal muscle aging lead to ion disturbances that impact fatigue and strength [165,168,169].

Calcium plays an important role in muscle contractility, interacting directly with troponin C and indirectly with tropomyosin. This contact is required for myosin proteins to bind to actin filaments. Significant changes in calcium levels and management, including a decrease in the time it takes intracellular calcium transients to exit the SR, begin in late middle age [165]. Furthermore, the mitochondrial calcium uniporter is disrupted, and the muscle’s response to calcium levels is diminished [102,139,165].

These changes are most prominent after 20 months of age in rodents, with reduced peaks of intramuscular calcium [165,170,171] and calcium uptake by SR [165,168,172] and mitochondria [139] but increased intramuscular level disturbances [143,173]. These alterations have been linked to reduced tetanic contraction in mice [168].

**Figure 5:**
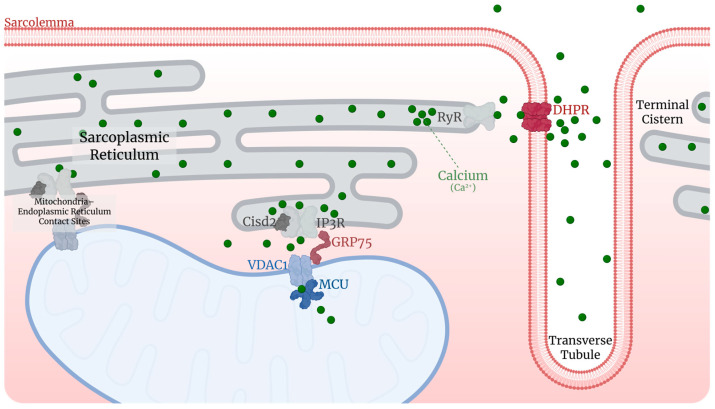
Proteins responsible for physiological calcium regulation are associated with skeletal muscle aging. Alterations in the mitochondrion–endoplasmic reticulum contact sites, Mitochondrial Calcium Uniporter complex (MCU), RyR and dihydropyridine receptor (DHRP) are among the features that lead to a diminished response to calcium and disturbed contraction with aging.

Part of the impairment associated with aging appears to be linked to the ryanodine receptor (RyR) in the SR, as its protein content decreases with age (Figure 5) [174]. This includes a significant reduction in the activity of RyR1, which is responsible for calcium release, with notably diminished activity in mice older than 20 months [173]. Consequently, the release of calcium from the sarcoplasmic reticulum (SR) is altered due to aging, directly affecting the production of ex vivo muscle force [174]. As a result, the maximum force produced by muscle declines with age, as demonstrated in the flexor digitorum brevis (FDB) muscle of both young (3–6 months) and old (20–22 months) mice [168]. Some of these changes can be ameliorated through treatment, such as the overexpression of insulin-like growth factor 1 (IGF-1) in transgenic mice, which enhances the expression of the *α1s* gene, which encodes the calcium channel dihydropyridine receptor (DHPR) [170].

The close relationship between mitochondria and the SR also plays an important role, as shown by reduced mitochondria-sarcoplasmic reticulum contact coverage (MERC) and increased thickness in mice at 18 months, leading to reduced calcium function and impacting mitochondrial enzymes that use it as a cofactor [102,175]. One protein present in the SR and mitochondria that influences calcium homeostasis is Cisd2. The presence of this mitochondrial protein abruptly declines by up to 66% from 3 to 12 months of age in mice [176,177]. Although there is no clear data about when this reduction starts, it apparently precedes early middle age [176,177]. Similarly, the protein EMRE is a uniporter complex whose activity has been reported to be reduced by up to 75% in 18 mo mice when compared to 6 mo animals [139].

Finally, the presence of the protein MICU3 from the Mitochondrial Calcium Uniporter (MCU) complex decreases with age, demonstrating mitochondrial inefficiency in calcium absorption and metabolism [139]. It is worth noting that overexpression of an inhibitory protein in this complex, MCUb, results in a shift in substrate preference from glucose to fatty acid and, eventually, lower intramuscular lipid levels [178]. A similar effect can be observed in MCU-deficient mice; however, it is unclear whether these effects are due to a natural drop in MCU activity with aging or an adaptive response [179].

The imbalance in ion homeostasis is not limited to calcium. Aging leads to an increase in levels of intramuscular sodium ions at rest [143,180], while magnesium levels decrease [181]. In elderly rats, the muscular conductance of chloride ions decreases, whereas potassium conductance increases [182]. Furthermore, FDB muscles have decreased channels of active potassium transport and elevated potassium-calcium levels [183].

Lastly, ionic iron accumulates over time and is directly related to glucose metabolism, oxidative distress, and circadian clock dysregulation [182,184]. Indeed, cases of hemochromatosis are linked with the promotion of diabetes mellitus II, while dysregulated circadian clocks can lead to muscle atrophy [185]. Hence, the significant accumulation of iron observed in the skeletal muscle of at-least-18-month-old rats—concomitant with type IIB fiber atrophy—may play a complex role in the exacerbated muscle decline in old age [182].

## 4. Structural Adaptations

**Figure 6:**
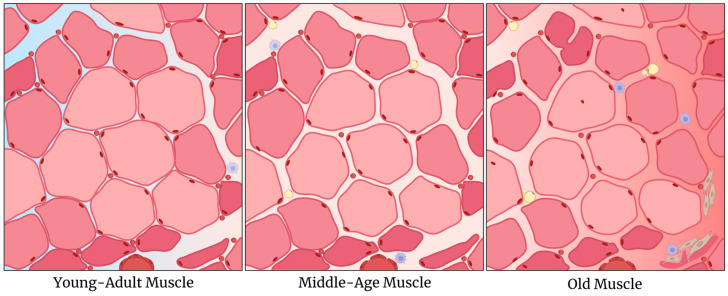
Structural changes in skeletal muscle throughout the aging process. Muscle aging is followed by progressive increase in fiber atrophy, increased fiber shape deformation, fibrosis, infiltration of non-muscle cells (adipocytes, fibroblasts, and macrophages), reduced number of vessels and vessels size, and loss of structural organization. Alterations are initially present in type IIB fibers, while during old age, they spread to all types of muscle fibers.

After reaching peak muscle mass and strength at around the age of 30, individuals experience an initial decline in skeletal muscle mass of up to 1% per year [186,187]. This decline follows an exponential pattern, leading to accelerated and non-linear muscle mass loss, particularly after middle age, when the loss becomes progressive. In mice, rats, and humans, the muscle atrophy begins at a slower rate and primarily affects glycolytic fibers [188,189,190]. Research shows that this decline typically starts between 12 and 15 months of age [19,51,87,102,123,140,191,192]. This process results in a shift in the fiber population, increasing the presence of oxidative fibers, which appear to be more resistant to aging (Figure 6) [188]. Significant mass loss in these oxidative fibers is typically observed only after animals reach 22 months of age, as evidenced by changes in soleus muscle weight [18,21,122,193,194].

These alterations are concomitant with a reduction in cross-sectional area [19,51,87,97,102] and an increase in density [19,191], but they are preceded by a decline in the ratio of muscle mass normalized by body weight—which begins between the ages of 8 to 14 mo in some muscles [33,97,191]. In humans, similar declines in cross-sectional area and strength relative to body mass index are visible in subjects between 30 and 50 years old or older [189,195].

Indeed, muscle mass and strength are correlated with age [32,78]. Together, both represent the key features for the development of sarcopenia—a late-stage disease that reduces mobility and life quality, affecting around 15–20% of people more than 60 years old [78,196]. Furthermore, muscle fiber aging is also associated with the loss of vascular, neuromuscular, and immune functions.

### 4.1. Myonuclear Architecture

One morphological marker of muscle regeneration is the presence of central nuclei, which indicates fiber remodeling through the incorporation of satellite cells. As aging progresses, myonuclear accretion increases to compensate for the loss of myonuclei and the cumulative damage in the sarcoplasm [78,192]. A reduced number of myonuclei, a lower number of myonuclei per fiber, and a smaller satellite cell pool have been reported in mice and rats at as early as 13–15 months of age [19,63,140,192]. The increase in central nuclei reflects a more vigorous attempt of the muscle to restore myonuclear presence and counteract sarcoplasma and genetic damage [14]. Although central nuclei indicate regeneration and have physiological roles such as maintaining muscle after exercise, their persistence with aging also suggests an effort of the muscle to contain increasing damage.

The consequence of increased myonuclear accretion over time is greater variability in myonuclear domain (MND) size. Type IIB fibers have a larger MND than type I fibers and show reduced protein turnover, as they require less protein synthesis (Figure 2) [14,197]. The broader cytoplasmic area governed by each myonucleus in type IIB fibers may explain their greater susceptibility to aging, since their limited protein turnover makes them less resilient to myonuclear loss [188,197].

Aging-related changes in the MND reflect altered myonuclear dynamics, including regeneration and transcriptional activity, which are crucial for fiber maintenance. For example, hypertrophy is associated with satellite cell mobilization and subsequent myonuclear accretion, adjusting the myonuclear number to match the increased fiber cross-sectional area [198,199,200,201]. In contrast, during atrophy, the regulation of myonuclear content to preserve MND size appears less flexible, with reductions in myonuclear number reported in up to 30% of cases [202]. This reduced adaptability is also evident with aging, wherein defects in satellite cell activation and mobilization and abnormal myonuclear content, particularly in sarcopenia, are well established [19,53,63,140,192].

Interestingly, the reduction in muscle fiber cross-sectional area appears to be linked to an increase in MND size, which becomes more pronounced in older animals (at 16–26 months old), particularly in larger fibers [191,192,197]. This phenomenon has been observed in muscles such as the tibialis anterior, gastrocnemius, and extensor digitorum longus (EDL) in mice, all of which contain a substantial proportion of type IIB fibers [191,192,197]. In rats, some studies showed a reduction in the size of the MND in IIA fibers of the soleus muscle [203], while no changes were observed in the diaphragm around 24 months [204]. These findings suggest that MND alterations with aging are dependent on both muscle type and species. Notably, the rat MND resembles that of humans, who show a well-established decline around the age of 60 [205,206], accompanied by increased variability in MND size [205].

In this context, alterations in the MND and the loss of functional myonuclei due to cumulative injury with age may significantly contribute to muscle mass decline. This reduction in mass leads to impaired protein turnover, which compromises both the maintenance and plasticity of muscle fibers [197,202,207]. Alarmingly, even surviving healthy myonuclei exhibit molecular alterations, including microtubule disorganization, which increases the entropy of nuclear spatial organization within the muscle fiber [192,198].

### 4.2. Muscle Vascularization and Oxygen Supply

Alterations in capillarization significantly impact nutrient delivery and endocrine communication in skeletal muscle, negatively contributing to the already abnormal metabolism observed in old age. Changes in capillary and vessel parameters are closely correlated with factors such as fiber size, myonuclear content, the myonuclear domain, and satellite cell proliferation [208,209].

During development, an increase in muscle vascularization to support muscle growth is evidenced by the number of capillaries per fiber. However, capillary densities decline until rodents reach approximately 9 months of age [210,211]. Likewise, aging animals display a reduction in vessel size and the number of capillaries per fiber [208,212]. Furthermore, not only is the muscle’s ability to enhance blood supply affected in rodents aged 9 to 20 months, but parameters related to oxygen uptake and consumption are also downregulated in both mouse [96] and rat [30] models.

Humans exhibit early alterations in vascular function and oxygen parameters as they age. For instance, by the age of 35, the minimum oxygen saturation and recovery time after exercise are already negatively impacted [89]. During this period, there are observable reductions of up to 20% in the vessel size of the orbicularis oculi muscle and a 30% decrease in vessel coverage by pericytes [213]. Additionally, research suggests that pericyte transplantation could be a potential therapeutic approach to restoring capillary maintenance in cases of muscle disuse [214].

Once individuals reach the age of 40, these trends become even more pronounced. By 47 years old, oxygen saturation levels are reduced not only at rest [89] but also during exercise, accompanied by increased levels of deoxyhemoglobin [215]. In certain muscles, such as the lateral great muscle, vessel thickness may decrease by as much as 50% in 48-year-olds [213]. Furthermore, in older age, the basement membrane of capillaries becomes thicker, which impairs nutrient delivery [216,217].

### 4.3. Neuromuscular Deterioration

**Figure 7:**
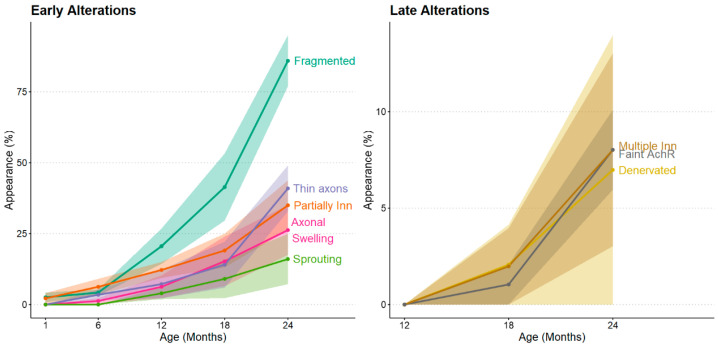
Timeline of neuromuscular aging. Based on the work of Gregório Valdez et al. (2010) [223]. Early alterations, gradually increasing after 6 months in mice, include fragmented AChRs, thinner axons, signs of partial innervation, axonal swelling, and sprouting of new axonal branches. Late alterations, appearing after 12 months in mice, include multiple innervations, faint AChRs, and signs of denervation.

Muscle impairment is closely associated with the aging of the nervous system, which coordinates the functionality of skeletal muscle. For example, SIRT1 mediates central circadian control in the suprachiasmatic nucleus through Bmal1 and CLOCK, whose activity otherwise declines with age [218]. The inhibition of circadian control in the brain is linked to mitochondrial dysfunction, muscle atrophy of type IIB fibers, and the accelerated onset of sarcopenia via Bmal1 expression in mice [185]. This relationship between brain function and muscle performance is significant. Alterations in neurocortex motor synapses and cortico-muscular coherence have been observed as early as age 40 in humans [219]. Similarly, in middle-aged mice, changes in motor nerve synapses and reduced latency have been observed in the diaphragm, indicating an adaptive response that increases action potential at the onset of skeletal muscle aging (Figure 7) [99].

Several mechanisms work to maintain the integrity of the neuromuscular junction (NMJ) until significant alterations appear in the later stages of aging. The onset of NMJ degeneration seems to occur at as early as 12 months of age in mice [220,221,222,223]. Over time, coverage of the NMJ is lost, along with the alignment between synaptic terminals and postsynaptic receptors, as evidenced by a reduced overlap between presynaptic and postsynaptic sides [221,222,224]. This period marks the beginning of alterations in the motor endplate, although the diameter of the motor axon remains intact. This suggests that the postsynaptic components are more susceptible to the aging process than the presynaptic ones [220].

As aging progresses, the frequency of AChR (acetylcholine receptor) fragments at the motor endplate gradually increases until 19 months of age, at which point up to 30% of the AChR area has already been lost [224]. In response to this loss, Schwann cells are mobilized to repair the damaged neuromuscular junctions (NMJs) and actively engage in their cytoplasmic processes to support the formation of axonal sprouts during denervation [225]. Additionally, the presence of migratory Schwann cells—which are associated with nerve regeneration—increases by more than 25% in the extensor digitorum longus (EDL) muscle of 17-month-old mice, indicating the organism’s efforts to counteract the loss of innervation [225].

Interestingly, this period at 19 months coincides with the emergence of ionic changes and mitochondrial abnormalities [220,224]. Mitochondrial dysfunction plays a significant role in the decline of NMJs, as mitochondria cluster around these junctions like myonuclei. Consequently, NMJs are particularly sensitive to excessive quantities of free radicals and the ineffective uptake and release of calcium due to defective mitochondria [226]. At this stage, there is also a noticeable reduction in axonal diameter and myelin thickness [193,208].

Proprioceptive sensory neurons found in muscle spindles are also affected by aging. In the early stages of aging in the extensor digitorum longus (EDL) muscle, specifically at 12 months, there is a reduction in the number of sensory neurons. This loss becomes more significant after 15 months [227]. Although the size of the intramuscular fibers remains unchanged, there is an increase in denervation, while the innervation slightly decreases [223,228]. Additionally, the proprioceptive sensory neurons exhibit thinner axons, a higher incidence of Ia afferents unraveling from the equatorial region, larger end plates, and fragmented acetylcholine receptors (AChR) [227,228].

The degradation of neuromuscular junctions (NMJs) is characterized by molecular changes, such as alterations in the expression of genes related to NMJ structure and function. These changes indicate a decompensation involving protective proteins [229,230]. Notably, advanced age disrupts the Agrin-MuSK-Lrp4 signaling pathway, which is essential for maintaining NMJ integrity and function. Reduced levels of Agrin can be detected in the tibialis anterior and EDL of mice aged 12 to 18 months, while alterations in the soleus muscle become apparent at 18 to 24 months [231]. Furthermore, investigations with Agrin knockout mice associated low levels of the protein with a loss of lean mass, muscle strength, and satellite cell activity [231].

In contrast, MuSK levels increase with age in both rodents and humans [21,229] and have been implicated in the maintenance of Akt-mTOR signaling in slow oxidative muscle [232]. The association of these biomarkers with neuromuscular junction (NMJ) aging is supported by knockout models of Agrin, Lrp4, MuSK, ERK1/2, and trkB, which result in synaptic changes similar to those seen in aging [226,231,232]. Similarly, deficiencies in laminin α4, Megf10, or the Erm transcription factor also lead to postsynaptic fragmentation [230,233,234].

Like many aspects of the aging process, the loss of motoneurons is not uniform. Peripheral neurons associated with fast-twitch fibers are more susceptible to damage, and their distal axonal segments are particularly vulnerable to disruptions, including those in axonal transport [193]. One significant issue affecting axonal transport is microtubule glycation, which becomes more pronounced in older individuals, especially due to their reduced insulin sensitivity and increased basal glycemia [235,236]. Additionally, glucose modifications occur in the membranes of these neurons, impairing neural signaling and permeability.

### 4.4. Immune Aging in Muscle

The complexities associated with middle age, such as increased body weight and reduced lean muscle mass, significantly impact inflammatory and immune parameters. These changes directly influence skeletal muscle regeneration following both chronic and acute injuries. After midlife, the homeostatic balance of immune cells is notably altered, with an increase of up to 50% in CD45+ cells, including macrophages and neutrophils [20,237,238,239].

Phenotypically, macrophages in aged muscle display a more pro-inflammatory profile, with reduced polarization toward the M2 subtype at both injured and non-injured sites [212,237,238,239,240]. One key mechanism impairing the M1-to-M2 immunomodulatory transition is the decline in MANF and selenoprotein P expression in macrophages during muscle regeneration [212,239]. Consequently, pro-inflammatory cytokines such as IL1β, IL12b, and IFNγ remain chronically elevated, contributing to the development of sarcopenia [237,238,241,242,243].

At the transcriptional level, aging is associated with the upregulation of NF-κB–driven inflammatory genes, including *CD14*, *Ccl2*, and *ASC*, which further promote the pro-inflammatory muscle phenotype [17,24,48,244]. In contrast, MAST2 and INPPL1, both negative regulators of NF-κB signaling, are downregulated in aged muscle [17]. This expression pattern reinforces the inflammatory skewing of the aged muscle microenvironment. Long-term transcriptional shifts linked to inflammation also become evident in humans, with pathways associated with immune activation and cell proliferation significantly altered starting in the fourth decade of life and extending into the fifth decade [3].

Another study demonstrated that IFNγ, together with TNFα, favors the differentiation of satellite cells over their proliferation during the later stages of muscle repair [212,238]. In parallel, the presence of macrophage subclusters associated with ECM remodeling further drives satellite cells toward a fibrogenic fate, ultimately promoting fibrosis [237,238,243]. Muscle fibrosis remains minimal and not statistically significant until approximately 14 months of age but becomes markedly evident between 17 and 19 months of age in rodents [87,94,237].

Other immune cell populations are also altered during muscle aging, like the T and B lymphocytes, which have been reported to decline by 50% and 30%, respectively [237]. However, divergent findings indicate increased levels of B and T cells in aged muscle [238,239]. While the basal abundance of adaptive immune system cells remains unclear, impaired recruitment and functionality of T and B cells in response to muscle injury are better documented [239,245].

Ultimately, these immune alterations contribute to the gradual accumulation of necrotic fibers and cellular debris, allowing tissue stressors and damaging stimuli to persist for prolonged periods [239]. Throughout life, skeletal muscle relies on lipids and oxylipins to regulate inflammation and maintain tissue homeostasis. Yet, in individuals of advanced age—such as octogenarians—these pathways appear to be more heavily mobilized, likely reflecting an adaptive response to chronic low-grade inflammation that leads to exhaustion of pro-resolving mediators [246]. Among the changes, omega-3 oxidation pathways stand out as markedly upregulated, orchestrating a broad anti-inflammatory program that also supports vascularization and hemostasis in sarcopenic individuals [247]. This remodeling of lipid metabolism in older muscle is accompanied by a measurable decline in circulating and intramuscular pools of polyunsaturated fatty acids, as reported for aged humans and experimental animal models [128].

## 5. Advanced-Age Skeletal Muscle

After approximately 75 years in humans and 25 months in rodents, skeletal muscle exhibits a marked accumulation of mechanical damage resulting from contraction and external stressors. These deleterious stimuli surpass the capacity of molecular adaptations, which are fully mobilized during mid-life, necessitating drastic responses such as highly active regeneration mediated by satellite cell mobilization. However, satellite cells display functional exhaustion and instability, with their pools undergoing pronounced molecular alterations, including depletion of glutathione reserves [53]. As empty niches can no longer be replenished with functional myofibers, fibrotic processes are activated to partially preserve tissue structure, albeit at the expense of contractile functionality. At this stage, even exercise interventions are insufficient to fully restore tissue integrity or completely recover prior muscle robustness [187].

## 6. Search Method Strategy

This review was conducted as a narrative synthesis of the literature focusing on early molecular and cellular alterations during skeletal muscle aging. Relevant articles were identified by searching the PubMed, Web of Science, and Google Scholar databases for records up to August 2025. The search strategy included combinations of the following terms: “skeletal muscle aging”, “early aging”, “midlife”, “middle-age”, and “biomarkers”, “sarcopenia onset”.

According to the inclusion criteria, we included

(i) Original experimental studies reporting molecular, metabolic, or structural changes in skeletal muscle associated with aging;
(ii) Studies focusing on young adults and middle-aged rodents or humans;
(iii) Reviews that provided relevant mechanistic insights.

We excluded

(i) Studies exclusively comparing young vs. very old/sarcopenic groups without addressing early alterations;
(ii) Studies that did not involve mouse, rat, or human models.

References were then organized according to the ages of the groups and thematic categories, including myonuclear changes, genetic and epigenetic regulation, mitochondrial and metabolic adaptations, oxidative stress, structural remodeling, and immune or neuromuscular alterations.

## 7. Conclusions

There is a paucity of evidence regarding middle age in animal models, particularly concerning skeletal muscle. The absence of data on this life stage in animal models is a challenge for the field of aging, conflating the causal changes in aging with cellular adaptations. Consequently, our analysis encapsulates the majority of experimental studies examining the onset of skeletal muscle deterioration and correlates the identified changes with recognized biomarkers of advanced muscle aging, including characteristics of sarcopenia. By concentrating on linear and non-linear biomarkers, we aim to assist laboratories in refining their studies and more accurately identifying the underlying physiological problems that result in a gradual deterioration of muscular performance over time.

## Abbreviations

The following abbreviations are used in this manuscript:

AChR	Acetylcholine receptor
AP-1	Activator protein 1 (FOS/JUN heterodimer transcription factor)
ASC	Apoptosis-associated speck-like protein containing a CARD
ATF4	Activating transcription factor 4
ATP	Adenosine triphosphate
Bmal1	Brain and muscle ARNT-like 1
CAT	Catalase
Ccl2	C-C motif chemokine ligand 2
CETN2	Centrin-2
CLOCK	Circadian locomotor output cycles kaput
CoQ	Coenzyme Q
CPT1B	Carnitine palmitoyltransferase 1B
CS	Citrate synthase
CuZnSOD	Copper-zinc superoxide dismutase (cytosolic)
CYP26B1	Cytochrome P450 family 26 subfamily B member 1
DHPR	Dihydropyridine receptor (calcium channel)
DRP1	Dynamin-related protein 1
EDL	Extensor digitorum longus
EMRE	Essential MCU regulator
ENO3	Enolase 3
ERK1	Extracellular signal-regulated kinase 1
EZH2	Enhancer of zeste homolog 2
FABP3	Fatty-acid-binding protein 3
FDB	Flexor digitorum brevis
FIS1	Mitochondrial fission 1 protein
FoxO	Forkhead box O
GLUT4	Glucose transporter type 4
GPx	Glutathione peroxidase
GPCPD1	Glycerophosphocholine phosphodiesterase 1
GR	Glutathione reductase
GSH	Glutathione reduced
GSK3	Glycogen synthase kinase 3
GS	Glycogen synthase
GSSG	Glutathione oxidized
HDAC	Histone deacetylase
H2A.Z	Histone variant H2A.Z
H3.1	Histone H3.1
H3.2	Histone H3.2
H3.3	Histone H3.3
HFD	High-fat diet
HK	Hexokinase
HNE	4-hydroxynonenal
ICAM	Intercellular adhesion molecule
IDH	Isocitrate dehydrogenase
IFNγ	Interferon gamma
IGF-1	Insulin-like growth factor 1
IKK	IκB kinase
IL6	Interleukin 6
IKB	Inhibitor of NF-κB
INPPL1	Inositol polyphosphate phosphatase like 1 (SHIP2)
IRS	Insulin receptor substrate
JNK	c-Jun N-terminal kinase
KGDH	Alpha-ketoglutarate dehydrogenase
Ku80	XRCC5 DNA repair protein (Ku heterodimer subunit)
LDH	Lactate dehydrogenase
LMNA	Pre-lamin-A/C
Lrp4	Low-density lipoprotein receptor-related protein 4
MAF	Musculoaponeurotic Fibrosarcomatranscription factor family
MANF	Mesencephalic astrocyte-derived neurotrophic factor
MAST2	Microtubule associated serine/threonine kinase 2
MCAM	Cell adhesion molecule (mentioned in context of muscle aging)
MCU	Mitochondrial calcium uniporter
MCUb	Mitochondrial calcium uniporter dominant-negative subunit b
MEF2	Myocyte enhancer factor 2
MERC	Mitochondria–sarcoplasmic reticulum contact coverage
Mfn	Mitofusin
MHC	Myosin heavy chain
MICOS	Mitochondrial contact site and cristae organizing system complex
Mll2	KMT2B—Lysine methyltransferase 2B
MND	Myonuclear domain
MnSOD	Manganese superoxide dismutase (mitochondrial)
mo	Months-old
MRE11	MRE11 homolog, double-strand break repair nuclease
MTF2	Metal response element-binding transcription factor 2
MuSK	Muscle-specific kinase
NAD+	Nicotinamide adenine dinucleotide (oxidized form)
NADH	Nicotinamide adenine dinucleotide (reduced form)
NF-κB	Nuclear factor kappa B
NMJ	Neuromuscular junction
NRF2	Nuclear factor erythroid 2-related factor 2
Opa1	Mitochondrial Dynamin Like GTPase; also known as ‘Optic atrophy 1’
PARP-1	Poly(ADP-ribose) polymerase 1
PCDHG	Protocadherin gamma cluster
PDH	Pyruvate dehydrogenase
PDK1	Phosphoinositide-dependent kinase 1
PDK2	Phosphoinositide-dependent kinase 2
PFKM	Muscular phosphofructokinase
PGAM2	Phosphoglycerate mutase 2
PGK1	Phosphoglycerate kinase 1
PGM1/2	Phosphoglucomutase 1 and 2
PI3K	Phosphoinositide 3-kinase
PIP3	Phosphatidylinositol (3,4,5)-trisphosphate
PK	Pyruvate kinase
PKM1	Pyruvate kinase muscle isozyme 1
Pofut1	Protein O-fucosyltransferase 1
ROS	Reactive oxygen species
RyR	Ryanodine receptor
SDH	Succinate dehydrogenase
SIRT	Sirtuin
SOD	Superoxide dismutase
SR	Sarcoplasmic reticulum
TNFα	Tumor necrosis factor alpha
VLCAD	Very-long-chain acyl-CoA dehydrogenase
WRN	Werner syndrome helicase
